# Association between predicted body composition and metabolic-associated fatty liver disease: a case-control study in a Chinese population

**DOI:** 10.3389/fmed.2026.1750432

**Published:** 2026-06-10

**Authors:** Shuang Liang, Mingli Li, Jiali Lin, Shengzhu Huang, Yulan Bai, Zefeng Chen, Xinyang Long, Yuanfan Li, Qianqian Luo, Zheng Wen, Zengnan Mo

**Affiliations:** 1 Department of Postgraduate and Continuing Education, The First Affiliated Hospital of Guangxi Medical University, Nanning, Guangxi, China; 2 Center for Genomic and Personalized Medicine, Guangxi Key Laboratory for Genomic and Personalized Medicine, Guangxi Collaborative Innovation Center for Genomic and Personalized Medicine, Guangxi Medical University, Nanning, Guangxi, China; 3 Research Center of Health Management, Guangxi Zhuang Autonomous Region People’s Hospital, Guangxi Academy of Medical Sciences, Nanning, Guangxi, China; 4 Department of Hospital Infection Management, Guangxi Medical University College of Stomatology, Nanning, Guangxi, China; 5 Department of Scientific Research and Discipline Construction, Guangxi Hospital Division of The First Affiliated Hospital, Sun Yat-sen University, Nanning, Guangxi, China; 6 Department of Hospital Infection Management, Wuming Hospital of Guangxi Medical University, Nanning, Guangxi, China; 7 Department of Hospital Infection Management, The People’s Hospital of Guangxi Zhuang Autonomous Region, Nanning, Guangxi, China; 8 Institute of Urology and Nephrology, First Affiliated Hospital of Guangxi Medical University, Guangxi Medical University, Nanning, Guangxi, China

**Keywords:** body composition, fat mass, lean body mass, metabolic-associated fatty liver disease, risk factor

## Abstract

**Objective:**

Metabolic-associated fatty liver disease (MASLD) is a growing global health concern. Few studies have examined the independent associations of fat mass (FM) and lean body mass (LBM) with MASLD. This study investigated the associations of predicted FM, body fat percentage (BF%), LBM, and the FM-to-LBM ratio with MASLD risk in a physical examination population from China.

**Methods:**

This 1:1 age- and sex-matched case-control study included 1,683 MASLD patients and 1,683 controls. Predicted FM, BF%, LBM, and FM/LBM were calculated using equations derived from the National Health and Nutrition Examination Survey (NHANES). Conditional logistic regression was used to analyze associations between body composition indices and MASLD risk after adjusting for confounders.

**Results:**

Levels of BMI, blood pressure, blood lipids, fasting glucose, liver enzymes, and uric acid were significantly higher in the MASLD group (all *P* < 0.001). The MASLD group also had significantly higher levels of FM, BF%, LBM, and FM/LBM (*P* < 0.001). After multivariable adjustment, the highest quartiles (Q4) of FM (OR = 2.77, 95% CI: 2.26–3.40), BF% (OR = 2.16, 95% CI: 1.78–2.62), LBM (OR = 1.62, 95% CI: 1.27–2.04), and FM/LBM (OR = 2.37, 95% CI: 1.99–2.83) were significantly associated with increased MASLD risk compared to the lowest quartiles (Q1) (all *P* < 0.001), with significant linear trends (*P* for trend < 0.001). Non-linear dose-response relationships were observed (*P* < 0.001). Subgroup analyses revealed stronger associations in individuals aged < 60 years, non-obese (BMI < 28 kg/m^2^), and non-hypertensive populations.

**Conclusion:**

Elevated levels of predicted FM, BF%, and FM/LBM were independently associated with increased MASLD risk, showing non-linear dose-response relationships. Higher predicted LBM was also associated with increased MASLD risk; however, this finding should be interpreted with caution. These associations were more pronounced in younger, non-obese, and normotensive individuals.

## 1 Introduction

Metabolic-associated fatty liver disease (MASLD), formerly known as non-alcoholic fatty liver disease (NAFLD), represents a spectrum of liver conditions, from simple steatosis to metabolic-associated steatohepatitis (MASH) (previously non-alcoholic steatohepatitis, NASH), fibrosis, and cirrhosis, occurring in the absence of significant alcohol consumption (1). The global prevalence of MASLD is estimated at 32.4% (2), imposing a substantial economic burden. Notably, China reports the highest number of prevalent cases and annual incident cases worldwide (3). MASLD is increasingly recognized not merely as a hepatic disorder but as a multisystem disease, strongly associated with extra-hepatic conditions including type 2 diabetes (T2D) and cardiovascular disease (CVD). Indeed, patients with MASLD are more likely to die from cardiovascular causes than from liver-related complications (4).

Metabolic-associated fatty liver disease is intrinsically linked to metabolic abnormalities, often considered the hepatic manifestation of metabolic syndrome (5). The pathogenesis has evolved from the classical “two-hit” hypothesis to a more complex, parallel multi-hit process involving multiple metabolic insults (6). Insulin resistance (IR) and chronic low-grade inflammation represent central mechanisms in its development.

Body Mass Index (BMI) remains commonly used for MASLD risk stratification despite its well-documented limitation in distinguishing between fat mass (FM) and lean body mass (LBM). This distinction is clinically relevant given that approximately 40% of MASLD cases occur in non-obese individuals (7). FM and LBM exert distinct and often opposing physiological influences: while dysfunctional adipose tissue promotes steatosis and inflammation (8), skeletal muscle serves as a major secretory organ and primary site of glucose disposal, significantly influencing IR and overall metabolic health (9). However, the independent roles of FM and LBM in MASLD pathogenesis remain incompletely understood, with epidemiological evidence presenting conflicting results (10). Some studies suggest a protective role for higher muscle mass (11), while others associate it with increased metabolic risk (12), indicating a complex relationship.

The interaction between FM and LBM remains underexplored in MASLD research. The FM-to-LBM ratio serves as a composite indicator that reflects the balance between metabolic burden (adiposity) and metabolic reserve (lean mass). An elevated ratio may indicate sarcopenic obesity, a state known to exacerbate insulin resistance and systemic inflammation – key drivers of MASLD. Indeed, this ratio has been associated with sarcopenic obesity, IR, and various metabolic disorders (13). However, few studies have directly examined the association between the FM/LBM ratio and MASLD risk, particularly in non-obese Asian populations. This gap motivated our investigation. Direct measurement of body composition via DXA or MRI is often impractical for large-scale epidemiological studies due to cost and logistical constraints. Prediction equations developed and validated by the National Health and Nutrition Examination Survey (NHANES) provide a reliable alternative for estimating FM, BF%, and LBM (14), with demonstrated associations to health outcomes including T2D and mortality (15, 16).

Therefore, this case-control study utilized a large-scale physical examination cohort from Guangxi, China, to investigate the independent and combined associations of predicted FM, BF%, LBM, and the FM/LBM ratio with MASLD risk, aiming to elucidate the specific contributions of body composition to MASLD development.

## 2 Materials and methods

### 2.1 Participants

This case-control study utilized baseline data from the Guangxi extension cohort of the “Community Population Cohort” project, part of a national key research and development initiative. The original cohort included 10,674 adults undergoing health check-ups at two participating hospitals between 2019 and 2020. All participants provided written informed consent, and the study protocol was approved by the Institutional Review Board of Guangxi Medical University.

**FIGURE 1:**
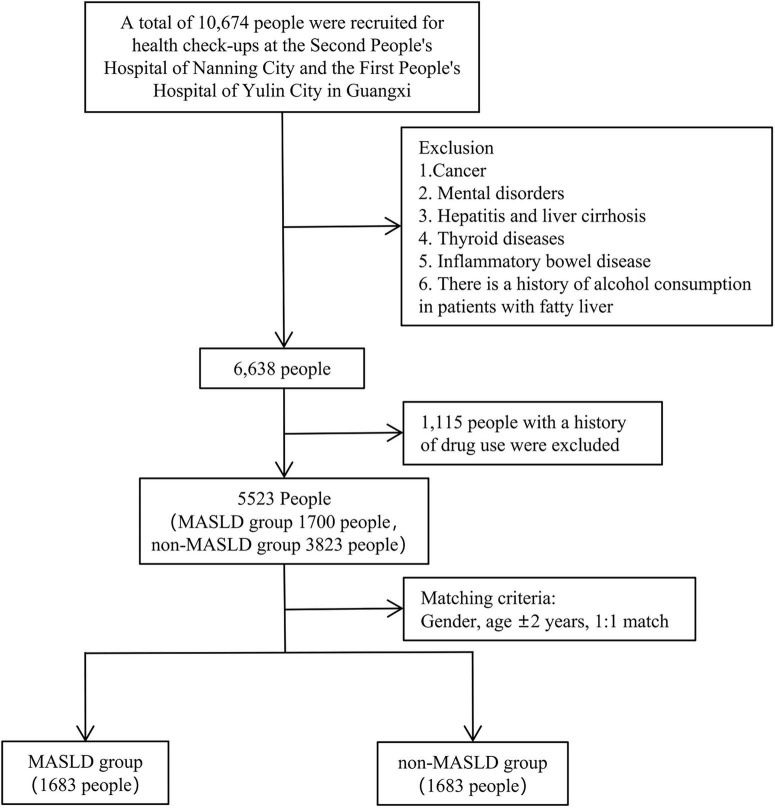
Flowchart of participant selection. After exclusions and matching, 3,366 participants were included.

Participants were excluded for the following reasons: history of cancer, mental or hematological diseases; viral or chronic hepatitis, cirrhosis, or other specific liver diseases; ulcerative colitis; hypothyroidism; significant alcohol consumption (≥140 g/week for men, ≥ 70 g/week for women); or absence of ultrasonography results. After applying exclusion criteria, 6,638 individuals remained. A further 1,115 individuals with a history of medication use affecting metabolic parameters were excluded, leaving 5,523 subjects (1,700 with MASLD and 3,823 without). From this pool, 1,683 MASLD cases were 1:1 matched with controls by gender and age (±2 years), yielding a final analytical sample of 3,366 participants (Figure 1).

### 2.2 Data collection

Trained personnel collected data through face-to-face interviews using a standardized questionnaire. Information gathered included socio-demographics (e.g., ethnicity, education level), lifestyle factors (smoking status, alcohol/tea consumption, physical activity, sedentary time), medication history, and family disease history. Smoking, alcohol consumption, and tea drinking were defined as habitual use for at least 6 months. Physical activity was categorized as engagement in any exercise lasting ≥ 10 min at least weekly.

Anthropometric measurements were conducted by medical staff following standardized protocols. Height and weight were measured using an ultrasonic stadiometer to calculate Body Mass Index (BMI). Blood pressure was measured twice with a mercury sphygmomanometer after a 15-min rest period, and the average was recorded. Fasting blood samples were analyzed for glucose, lipid profiles, liver enzymes (ALT, AST), and uric acid using automated biochemical analyzers.

### 2.3 Assessment of body composition

Fat mass (FM), lean body mass (LBM), and body fat percentage (BF%) were calculated using sex-specific prediction equations developed and validated by the NHANES study (14). These equations, which incorporate age, height, weight, and ethnicity, have demonstrated high agreement with DXA measurements (R^2^ ranging from 0.61 to 0.93) and strong correlations with obesity-related biomarkers. The FM-to-LBM ratio (FM/LBM) was subsequently computed (Supplementary Table 1).

### 2.4 Definitions of MASLD and metabolic conditions

At the time of study conduct, the disease was diagnosed as non-alcoholic fatty liver disease (NAFLD); it is now referred to as metabolic-associated fatty liver disease (MASLD). Accordingly, the diagnostic criteria were based on the Chinese guidelines for the management of non-alcoholic fatty liver disease (2010 updated edition) (17), requiring: (1) no history of significant alcohol consumption, defined as < 140 g/week for men and < 70 g/week for women; (2) exclusion of other specific liver diseases, including viral hepatitis, cirrhosis, hepatocellular carcinoma, drug-induced liver disease, total parenteral nutrition, Wilson’s disease, autoimmune hepatitis, hypothyroidism, and other conditions that can cause fatty liver; and (3) ultrasonographic evidence of diffuse fatty liver. Hyperlipidemia was defined as TG ≥ 1.7 mmol/L (18). Hyperglycemia was defined as fasting glucose ≥ 6.1 mmol/L, 2-h postprandial glucose ≥ 7.8 mmol/L, and/or a previous diagnosis of diabetes. Hypertension was defined as a prior diagnosis, use of antihypertensive medication, or measured SBP/DBP ≥ 140/90 mmHg without medication (19).

### 2.5 Statistical analysis

Continuous variables were described as mean ± standard deviation or median (interquartile range) and compared using *t*-tests or Mann-Whitney U tests, as appropriate. Categorical variables were described as frequencies and compared using chi-square tests.

Missing data were present for smoking history (*n* = 7, 0.21%), alcohol consumption (*n* = 6, 0.18%), tea drinking (*n* = 8, 0.24%), and weekly exercise (*n* = 30, 0.89%). In all cases, the proportion of missingness was less than 1%. Given the very low proportion and the assumption of missing at random, we performed a complete-case analysis.

Medication use (glucose-lowering, antihypertensive, lipid-lowering agents) was recorded but was too rare (<0.2% overall) to be meaningfully analyzed and was therefore not included as a covariate (Supplementary Table 7).

The associations between body composition indices (FM, BF%, LBM, FM/LBM) and MASLD were assessed using conditional logistic regression, accounting for the matched design. The indices were analyzed both as continuous variables and in quartiles (Q1-Q4), with Q1 as the reference. Trend tests were performed across quartiles. Multivariable models were adjusted for height, ethnicity, education, smoking, tea drinking, physical activity, sedentary time, hyperglycemia, hypertension, and uric acid. Mutual adjustment for FM and LBM was also performed. BMI was not included in the main models because it shares the same anthropometric basis (height and weight) as the predicted body composition indices; a sensitivity analysis adding BMI was performed. The dose-response relationships were examined using restricted cubic splines (RCS) with four knots.

Subgroup analyses were conducted by sex, age (<40, 40–60, ≥ 60 years), BMI (<24, 24- < 28, ≥ 28 kg/m^2^), hyperglycemia, and hypertension status. All analyses were performed using statistical software, with a two-sided *P*-value < 0.05 considered statistically significant.

## 3 Results

Baseline characteristics of the matched MASLD cases and controls are presented in Table 1. While groups were comparable in age and sex distribution, significant differences existed in lifestyle factors and comorbidities. The MASLD group reported longer sedentary time, lower rates of smoking and weekly exercise, and a higher prevalence of tea drinking (all *P* < 0.05). Cardiometabolic comorbidities, including obesity, hyperglycemia, hyperlipidemia, and hypertension, were substantially more prevalent in the MASLD group (all *P* < 0.001).

**TABLE 1:** Baseline characteristics of the study population.

Characteristics	Total population	Control group	MASLD group	*P*
Age (years)	41.84 ± 12.16	42.01 ± 11.93	42.00 ± 11.72	0.991
Gender				0.517
Male	2658 (79.0)	1329 (79.0)	1329 (79.0)	
Female	708 (21.0)	354 (21.0)	354 (21.0)	
Sedentary time(h)	5.43 ± 2.74	5.20 ± 2.71	5.66 ± 2.74	<0.001
Smoking history				<0.001
Yes	1034 (30.7)	586 (34.8)	448 (26.6)	
No	2325 (69.1)	1090 (64.7)	1235 (73.4)	
History of alcohol consumption				
Yes	442 (13.1)	442 (26.3)	0 (0.0)	<0.001
No	2918 (86.7)	1235 (73.4)	1683 (100.0)	
History of tea drinking				0.027
Yes	1234 (36.7)	585 (34.8)	649 (38.6)	
No	2124 (63.1)	1091 (64.8)	1033 (61.4)	
Exercise every week				0.024
Yes	2114 (62.8)	1089 (64.7)	1025 (61.0)	
No	1222 (36.3)	580 (34.5)	642 (38.1)	
Obesity				<0.001
Yes	439 (13.0)	55 (3.3)	384 (22.8)	
No	2927 (87.0)	1628 (96.7)	1299 (77.2)	
Hyperglycemia				<0.001
Yes	188 (5.6)	57 (3.4)	131 (7.8)	
No	3178 (94.4)	1626 (96.6)	1552 (92.2)	
Hyperlipidemia				<0.001
Yes	1217 (36.2)	349 (20.7)	868 (51.6)	
No	2149 (63.8)	1334 (79.3)	815 (48.4)	
Hypertension				<0.001
Yes	612 (18.2)	244 (14.5)	368 (21.9)	
No	2754 (81.8)	1439 (85.5)	1315 (78.1)	

Continuous variables are expressed as mean ± standard deviation, while categorical variables are expressed as quantity (n) and percentage (%).

As shown in Table 2, the MASLD group demonstrated significantly higher levels of BMI, blood pressure, blood lipids, fasting glucose, liver enzymes, and uric acid (all *P* < 0.001). All predicted body composition indices—FM, BF%, LBM, and FM/LBM—were significantly elevated in the MASLD group compared to controls (all *P* < 0.001).

**TABLE 2:** Comparison of biochemical indexes and body composition.

Characteristics	Total population	Control group	MASLD group	*P*
Height (cm)	165.93 ± 7.45	165.75 ± 7.31	166.10 ± 7.57	0.169
Weight (kg)	67.71 ± 11.25	63.19 ± 9.55	72.23 ± 11.01	<0.001
BMI (kg/m^2^)	24.51 ± 3.21	24.27 ± 3.65	27.48 ± 4.21	<0.001
Systolic blood pressure (mmHg)	127.11 ± 15.24	125.08 ± 15.34	129.14 ± 14.87	<0.001
Diastolic blood pressure (mmHg)	77.02 ± 10.71	75.70 ± 10.51	78.34 ± 10.75	<0.001
Total cholesterol (mmol/L)	4.96 ± 0.92	4.83 ± 0.89	5.08 ± 0.93	<0.001
Triglycerides (mmol/L)	1.75 ± 1.48	1.32 ± 0.97	2.18 ± 1.76	<0.001
High-density lipoprotein (mmol/L)	1.25 ± 0.30	1.34 ± 0.30	1.16 ± 0.26	<0.001
Low-density lipoprotein (mmol/L)	3.25 ± 0.85	3.14 ± 0.82	3.36 ± 0.86	<0.001
Fasting blood glucose (mmol/L)	5.03 ± 1.23	4.80 ± 0.93	5.16 ± 1.27	<0.001
Alanine aminotransferase (IU/L)	28.44 ± 22.77	23.01 ± 18.47	33.88 ± 25.25	<0.001
Aspartate aminotransferase (IU/L)	23.09 ± 10.75	21.79 ± 9.65	24.39 ± 11.60	<0.001
Creatinine (μmol/L)	80.60 ± 16.87	80.57 ± 16.65	80.62 ± 17.09	0.931
Uric acid (μmol/L)	395.95 ± 97.94	370.39 ± 90.59	421.30 ± 98.40	<0.001
FM (kg)	17.05 ± 5.13	17.05 ± 4.35	21.93 ± 4.67	<0.001
BF%	28.12 ± 4.87	25.94 ± 4.25	29.53 ± 4.77	<0.001
LBM (kg)	46.67 ± 8.11	45.53 ± 6.86	48.59 ± 8.48	<0.001
FM/LBM	0.43 ± 0.12	0.37 ± 0.10	0.46 ± 0.12	<0.001

Continuous variables are expressed as mean ± standard deviation, while categorical variables are expressed as quantity (n) and percentage (%). FM, fat mass; BF%, fat percentage; LBM, lean body mass; FM/LBM, The ratio of fat mass to lean body mass.

Conditional logistic regression analyses revealed significant associations between all body composition indices and MASLD risk (Table 3). A positive dose-response relationship was observed for FM across all quartiles in the crude model (Model 1). This association remained statistically significant after sequential adjustment for lifestyle factors (Model 2) and cardiometabolic conditions (Model 3), with the highest quartile (Q4) showing an OR of 2.77 (95% CI: 2.26–3.40) in the fully adjusted model.

**TABLE 3:** Hierarchical analysis of the correlation between FM, BF%, LBM, FM/LBM and MASLD.

Variables	Control group/MASLD group	Model1		Model 2		Model 3	
		OR (95%CI)	*P*	OR (95%CI)	*P*	OR (95%CI)	*P*
FMa							
Q1 ≤ 16.26	692/149	1.00 (ref.)		1.00 (ref.)		1.00 (ref.)	
Q2 (16.26–19.38)	491/351	2.62 (1.88–3.67)	<0.001	2.04 (1.68–2.49)	<0.001	1.93 (1.58–2.35)	<0.001
Q3 (19.38–22.61)	344/498	3.34 (2.78–4.01)	<0.001	2.64 (2.19–3.19)	<0.001	2.39 (1.97–2.91)	<0.001
Q4 > 22.61	156/685	4.60 (3.85–5.49)	<0.001	3.27 (2.71–3.93)	<0.001	2.77 (2.26–3.40)	<0.001
Trend test			<0.001		<0.001		<0.001
BF%							
Q1 ≤ 21.17	653/188	1.00 (ref.)		1.00 (ref.)		1.00 (ref.)	
Q2 (21.17–24.98)	402/390	2.07 (1.74–2.47)	<0.001	1.81 (1.51–2.16)	<0.001	1.66 (1.39–2.00)	<0.001
Q3 (24.98–29.27)	307/535	2.84 (2.41–3.36)	<0.001	2.21 (1.86–2.53)	<0.001	1.90 (1.58–2.28)	<0.001
Q4 > 29.27	271/570	3.03 (2.57–3.58)	<0.001	2.50 (2.10–2.97)	<0.001	2.16 (1.78–2.62)	<0.001
Trend test			<0.001		<0.001		<0.001
LBMa							
Q1 ≤ 41.33	490/350	1.00 (ref.)		1.00 (ref.)		1.00 (ref.)	
Q2 (41.33–47.69)	567/274	1.12 (0.94–1.34)	0.207	1.04 (0.86–1.26)	0.716	1.20 (1.00–1.45)	0.063
Q3 (47.69–52.17)	393/451	2.13 (1.78–2.55)	<0.001	1.62 (1.32–1.99)	<0.001	1.59 (1.30–1.94)	<0.001
Q4 > 52.17	233/608	3.60 (2.93–4.42)	<0.001	2.28 (1.80–2.89)	<0.001	1.62 (1.27–2.04)	<0.001
Trend test			<0.001		<0.001		<0.001
FM/LBM							
Q1 ≤ 0.35	656/185	1.00 (ref.)		1.00 (ref.)		–	–
Q2 (0.35–0.40)	425/417	2.25 (1.89–2.68)	<0.001	1.89 (1.58–2.26)	<0.001	–	–
Q3 (0.40–0.47)	275/567	3.06 (2.59–3.61)	<0.001	2.31 (1.94–2.74)	<0.001	–	–
Q4 > 0.47	327/514	2.78 (2.35–3.29)	<0.001	2.37 (1.99–2.83)	<0.001	–	–
Trend test			<0.001		<0.001		

Model 1 is a single-factor model; Model 2 adjusted for ethnicity, educational level, smoking history, drinking history, tea consumption history, weekly exercise, sedentary time, hypertension, hyperglycemia, and uric acid. Model 3 mutually corrected FM or LBM on the basis of Model 2. FM, fat mass; BF%, fat percentage; LBM, lean body mass; FM/LBM, The ratio of fat mass to lean body mass.

^a^Corrected the height.

A similar graded positive association was consistently observed for BF%. In contrast, the association between LBM and MASLD exhibited a distinct pattern, with significantly increased risk only apparent in the higher quartiles (Q3 and Q4) in the fully adjusted model. The FM/LBM ratio demonstrated a strong association with increased MASLD risk in both Models 1 and 2. All trend tests across quartiles were statistically significant (*P* for trend < 0.001). In a sensitivity analysis additionally adjusting for BMI, the associations of FM, BF%, and LBM with MASLD became statistically non-significant (all *P* > 0.05). However, the association of FM/LBM with MASLD remained significant (OR = 2.00, 95% CI: 1.43–2.78, *P* < 0.05; Supplementary Table 10).

**FIGURE 2:**
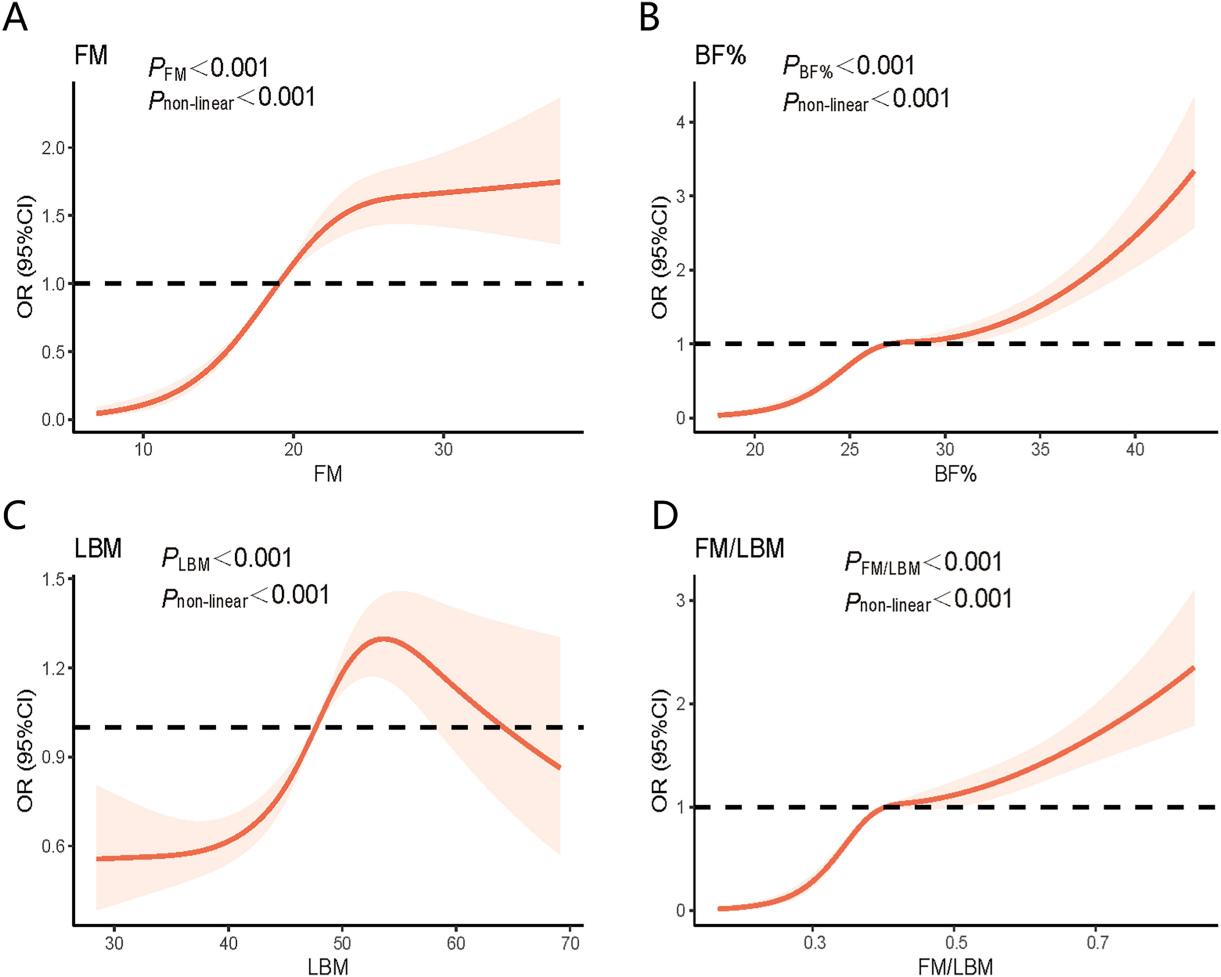
Nonlinear dose-response relationships between body composition indices and MASLD risk using restricted cubic splines. All associations showed significant nonlinear patterns (*P* < 0.001). **(A)** Fat mass (FM), **(B)** body fat percentage (BF%), **(C)** lean body mass (LBM), and (D) the FM/LBM ratio.

Restricted cubic spline analyses revealed significant non-linear relationships between all body composition indices and MASLD risk (*P* non-linear < 0.001, Figure 2). MASLD risk increased progressively with FM up to approximately 23 kg, after which it plateaued. For BF%, risk increased until approximately 27%, remained stable, then increased again. LBM showed an accelerating risk increase until approximately 53 kg, followed by a gradual decrease. The FM/LBM ratio demonstrated increasing risk until approximately 0.4, stabilization, and subsequent increase.

**FIGURE 3:**
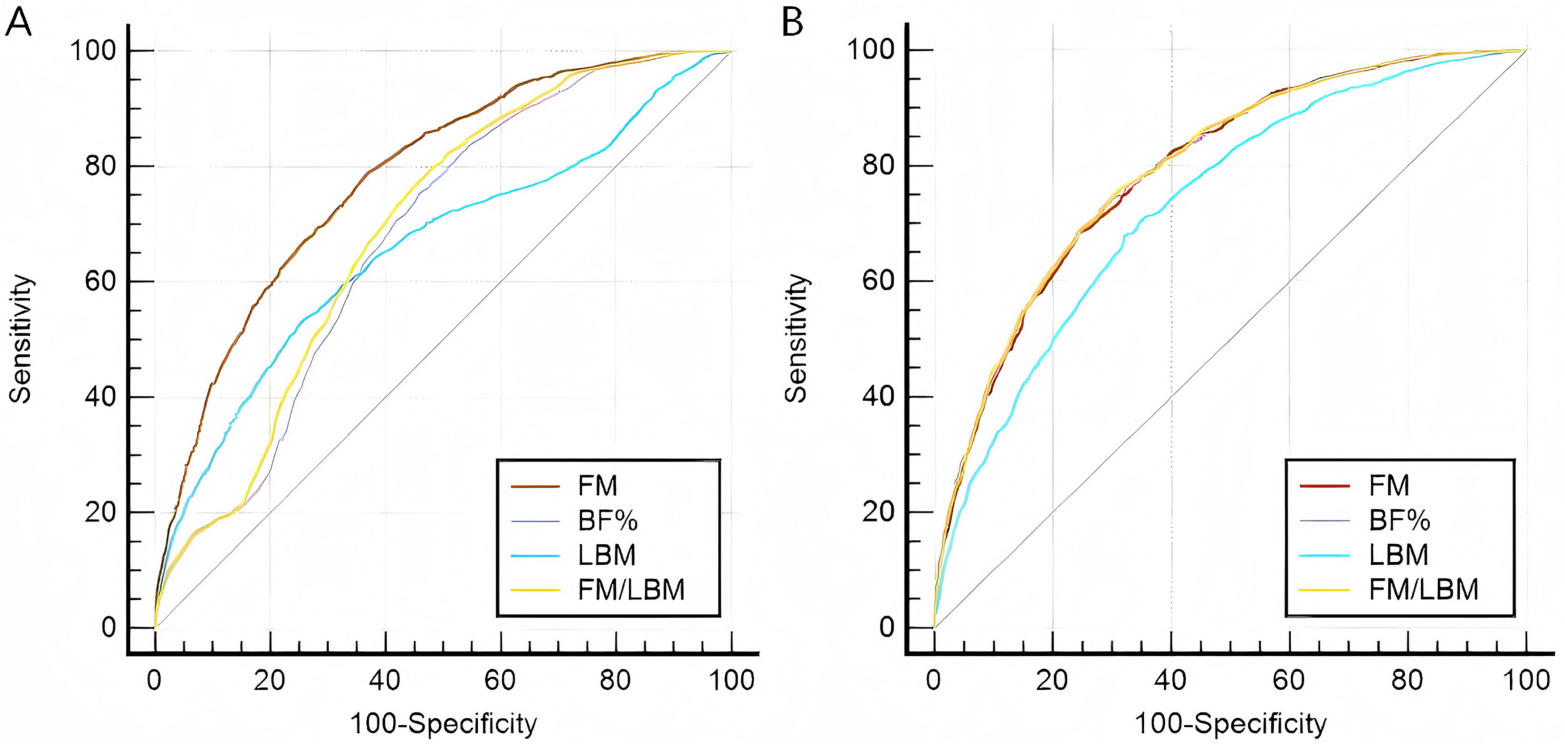
Receiver operating characteristic (ROC) curves for predicting MASLD. All curves rise above the diagonal reference line, indicating diagnostic value. Panel **(A)** shows the ROC curves for each index without adjustment; panel **(B)** shows the ROC curves for the same indices after adjustment for age and gender.

ROC analysis demonstrated that all individual indices had considerable predictive capability for MASLD (AUC range: 0.658–0.782), with FM showing the highest predictive value (AUC = 0.782, Table 4). In composite models incorporating age and gender, the predictive performance of all indices improved (AUC range: 0.734–0.795, Figure 3). Incremental predictive analysis showed that adding any of the predicted body composition indices (FM, BF%, LBM, or FM/LBM) to a base model containing age, sex, and BMI did not significantly improve the AUC for MASLD (all ΔAUC < 0.01, all *P* > 0.05; Supplementary Table 8). Subgroup-specific AUC analyses (Supplementary Table 9) showed that FM had AUCs of 0.702 (95% CI: 0.678–0.725) in non-obese individuals and 0.789 (95% CI: 0.732–0.839) in participants aged ≥ 60 years.

**TABLE 4:** The models for predicting the risk of MASLD with FM, BF%, LBM and FM/LBM.

Variables	AUC	95% CI	Sensitivity	Specificity	Youden index
Simple model					
FM	0.782	0.768–0.796	0.790	0.630	0.421
BF%	0.676	0.660–0.692	0.753	0.544	0.297
LBM	0.658	0.642–0.674	0.534	0.743	0.277
FM/LBM	0.691	0.675–0.706	0.826	0.491	0.317
Composite model					
FM	0.792	0.777–0.805	0.683	0.758	0.441
BF%	0.795	0.781–0.808	0.726	0.720	0.446
LBM	0.734	0.719–0.749	0.678	0.679	0.357
FM/LBM	0.794	0.780–0.808	0.746	0.702	0.449

FM, fat mass; BF%, fat percentage; LBM, lean body mass; FM/LBM, The ratio of fat mass to lean body mass.

Stratified analyses were performed according to sex, age, BMI, hypertension, and hyperglycemia status.

In non-obese individuals (BMI < 24 kg/m^2^), all body composition indices showed significant dose-response associations with MASLD (all *P* for trend < 0.001), with the highest quartile of BF% yielding an OR of 6.08 (95%CI: 3.78–9.80). In obese participants (BMI ≥ 28kg/m^2^), no significant associations were observed (all *P* for trend > 0.05; Supplementary Table 2).

Age stratification (Supplementary Table 3) showed that all indices were significantly associated with MASLD in participants aged < 60 years (both < 40 and 40–60 groups), but not in those aged ≥ 60 years (all *P* for trend > 0.05, except isolated quartiles).

Hypertension stratification (Supplementary Table 4) revealed significant associations for all indices in non-hypertensive participants (all *P* for trend < 0.001), whereas in hypertensive participants, only FM/LBM showed a significant trend (*P* = 0.023).

Positive associations were also observed across sexes (both males and females; Supplementary Table 5) and glycemic status (normoglycemic but not hyperglycemic; Supplementary Table 6).

## 4 Discussion

This large, matched case-control study demonstrates significant independent associations between predicted body composition indices and MASLD presence. Our principal findings indicate that higher quartiles of FM, BF%, LBM, and the FM/LBM ratio are associated with progressively increased odds of MASLD, following non-linear dose-response patterns. These relationships persist after comprehensive adjustment for lifestyle factors and cardiometabolic comorbidities, underscoring the fundamental role of body composition in MASLD pathophysiology.

The confirmed positive association between FM or BF% and MASLD aligns with established literature (20). A key methodological strength of our analysis is the mutual adjustment for body composition components. The robustness of the FM- and BF%-MASLD associations after controlling for LBM underscores adiposity as a primary, independent driver of the disease. This finding is further corroborated by our ROC analysis, which identified FM as the most predictive individual indicator. However, incremental predictive analysis showed that none of the predicted body composition indices significantly improved the AUC beyond a model containing age, sex, and BMI (all ΔAUC < 0.01, all *P* > 0.05; Supplementary Table 8). This suggests that, in our population, BMI alone has strong discriminative ability for MASLD. In a sensitivity analysis further adjusting for BMI, the associations of FM, BF%, and LBM with MASLD became non-significant due to overadjustment, whereas the FM/LBM ratio remained significantly associated (Supplementary Table 10). This is because FM/LBM reflects the balance between fat and lean mass, which is not fully captured by BMI alone. The persistence of significance for FM/LBM under this overadjusted model highlights its robustness as an independent risk marker for MASLD. Our primary model (Model 3) purposely excluded BMI to avoid overadjustment, and its findings remain valid. The observed risk plateau at higher FM levels (approximately 23 kg) may suggest a threshold effect.

Our findings present a more complex picture regarding LBM than previous reports. Contrary to studies suggesting a protective role for higher muscle mass (21), we found a positive association between LBM and MASLD. However, this finding should be interpreted with caution, as it may reflect confounding by overall body size, adiposity, or metabolic risk rather than a direct harmful effect of lean mass. Therefore, our results should not be misinterpreted as evidence that increasing muscle mass is harmful, but rather highlight the complexity of body composition in metabolic disease.

The FM/LBM ratio, an integrative metric reflecting the balance between energy storage and metabolic capacity, emerged as a strong, independent risk factor, consistent with its established role as a proxy for sarcopenic obesity and its documented associations with other metabolic disorders (13).

From a pathophysiological perspective, the link between excess FM and MASLD is likely mediated by adipose tissue dysfunction, which promotes systemic insulin resistance, chronic inflammation, and altered adipokine secretion, ultimately disrupting hepatic lipid and glucose homeostasis (22, 23). The mechanisms underlying the positive association with LBM remain unclear and warrant further investigation.

Our stratified analyses provide critical insights for clinical risk stratification. The pronounced associations of all body composition indices with MASLD in non-obese individuals highlight the clinical utility of these metrics for identifying “lean MASLD,” a substantial and often overlooked patient subgroup. Conversely, the attenuated or null associations in subgroups with obesity, hypertension, or advanced age suggest that in the context of established metabolic dysfunction or aging, other factors—such as visceral adiposity, profound insulin resistance, or declining muscle quality—may overshadow the predictive value of overall FM or BF%. The absence of association between LBM and MASLD in adults ≥ 60 years suggests that in older populations, muscle function may represent a more relevant metric than mass alone, consistent with modern sarcopenia definitions that prioritize strength and performance.

The cross-sectional nature of our study prevents causal inference, but bidirectional pathways are biologically plausible. On one hand, excess adiposity and low relative muscle mass may drive MASLD through insulin resistance and pro-inflammatory adipokines. On the other hand, MASLD itself, particularly in its more advanced stages, can promote systemic inflammation and alter amino acid metabolism, potentially accelerating muscle protein catabolism and leading to sarcopenia (24). This potential vicious cycle warrants investigation in prospective cohort studies.

This study has several limitations. First, the cross-sectional design precludes causal inference and the distinction between incident and prevalent MASLD. Second, MASLD was diagnosed by ultrasonography, which has reduced sensitivity for mild steatosis (hepatic fat < 20%) and cannot quantify fibrosis or disease severity; therefore, our findings primarily apply to ultrasonography-diagnosed MASLD. Third, body composition indices were estimated using prediction equations originally developed in the NHANES population, which may not be perfectly generalizable to Southern Chinese individuals; cautious interpretation is warranted, and future studies with direct measurements (e.g., DXA) are needed. Fourth, our questionnaire did not collect family history of diabetes or MASLD, and medication use (glucose-lowering, antihypertensive, lipid-lowering agents) was too rare (<0.2%) to analyze statistically. However, we adjusted for participants’ own hyperglycemia and hypertension, which partially account for metabolic risk. Fifth, we lacked data on visceral adipose tissue and muscle strength/function, which are important contributors to metabolic health. Despite these limitations, our study benefits from a large, well-phenotyped sample and a rigorous matched design, providing a comprehensive evaluation of multiple body composition indices simultaneously.

## 5 Conclusion

In conclusion, our findings affirm that predicted FM, BF%, and the FM/LBM ratio are significantly associated with MASLD, with these relationships being most salient in non-obese and younger-to-middle-aged adults. Higher predicted LBM was also associated with increased MASLD risk; however, this finding should be interpreted with caution, as it may reflect confounding by overall body size, adiposity, or metabolic risk rather than a direct harmful effect of lean mass *per se*. This observation underscores the complexity of body composition in metabolic disease and highlights the importance of methodological considerations in future research. Future prospective cohort studies and Mendelian randomization analyses are warranted to elucidate causality and to explore the potential of integrating these accessible body composition metrics into refined MASLD screening strategies, particularly for at-risk non-obese populations.

## Data Availability

The raw data supporting the conclusions of this article will be made available by the authors, without undue reservation.

## References

[1] Wang XJ Malhi H. Nonalcoholic fatty liver disease. *Ann Intern Med*. (2018) 169:ITC65–80. 10.7326/AITC201811060 30398639

[2] Riazi K Azhari H Charette JH Underwood FE King JA Afshar EE et al. The prevalence and incidence of NAFLD worldwide: a systematic review and meta-analysis. *Lancet Gastroenterol Hepatol*. (2022) 7:851–61. 10.1016/S2468-1253(22)00165-0 35798021

[3] Zeng J Gu C Wen C Shen C. The burden of NAFLD (now referred to as MASLD)-related chronic liver disease and cirrhosis from 1990 to 2021 with projections to 2036: a comparative study of global China the United States and India. *Lipids Health Dis*. (2025) 24:298. 10.1186/s12944-025-02750-z 41024003 PMC12482587

[4] Caussy C Aubin A Loomba R. The relationship between type 2 diabetes, NAFLD, and cardiovascular risk. *Curr Diab Rep*. (2021) 21:15. 10.1007/s11892-021-01383-7 33742318 PMC8805985

[5] Yki-Järvinen H. Non-alcoholic fatty liver disease as a cause and a consequence of metabolic syndrome. *Lancet Diabetes Endocrinol*. (2014) 2:901–10. 10.1016/S2213-8587(14)70032-4 24731669

[6] Fang YL Chen H Wang CL Liang L. Pathogenesis of non-alcoholic fatty liver disease in children and adolescence: from “two hit theory” to “multiple hit model”. *World J Gastroenterol*. (2018) 24:2974–83. 10.3748/wjg.v24.i27.2974 30038464 PMC6054950

[7] Ye Q Zou B Yeo YH Li J Huang DQ Wu Y et al. Global prevalence, incidence, and outcomes of non-obese or lean non-alcoholic fatty liver disease: a systematic review and meta-analysis. *Lancet Gastroenterol Hepatol.* (2020) 5:739–52. 10.1016/S2468-1253(20)30077-7 32413340

[8] Cariou B. The metabolic triad of non-alcoholic fatty liver disease, visceral adiposity and type 2 diabetes: implications for treatment. *Diabetes Obes Metab*. (2022) 24(Suppl 2):15–27. 10.1111/dom.14651 35014161

[9] Chiyanika C Wong VW Wong GL Chan HL Hui SCN Yeung DKW et al. Implications of abdominal adipose tissue distribution on nonalcoholic fatty liver disease and metabolic syndrome: a Chinese general population study. *Clin Transl Gastroenterol*. (2021) 12:e00300. 10.14309/ctg.0000000000000300 33600104 PMC7889374

[10] Ariya M Koohpayeh F Ghaemi A Osati S Davoodi SH Razzaz JM et al. Assessment of the association between body composition and risk of non-alcoholic fatty liver. *PLoS One*. (2021) 16:e0249223. 10.1371/journal.pone.0249223 33793621 PMC8016222

[11] Choi M Lee S Bae SH Chung S. Application of body composition zones in boys with nonalcoholic fatty liver disease. *Ann Pediatr Endocrinol Metab*. (2019) 24:243–7. 10.6065/apem.2019.24.4.243 31905444 PMC6944856

[12] Park HK Shim YS. Association between lean mass and the risk of metabolic syndrome in Korean children and adolescents: data from the Korea National Health and Nutrition Examination survey. *Endocrine J.* (2025) 72:1227–37. 10.1507/endocrj.EJ25-0178 40738623 PMC12624292

[13] Dai H Xiang J Hou Y Xuan L Wang T Li M et al. Fat mass to fat-free mass ratio and the risk of non-alcoholic fatty liver disease and fibrosis in non-obese and obese individuals. *Nutr Metab*. (2021) 18:21. 10.1186/s12986-021-00551-6 33608033 PMC7893940

[14] Lee DH Keum N Hu FB Orav EJ Rimm EB Sun Q et al. Development and validation of anthropometric prediction equations for lean body mass, fat mass and percent fat in adults using the National Health and Nutrition Examination Survey (NHANES) 1999-2006. *Br J Nutr*. (2017) 118:858–66. 10.1017/S0007114517002665 29110742

[15] Lee DH Keum N Hu FB Orav EJ Rimm EB Willett WC et al. Comparison of the association of predicted fat mass, body mass index, and other obesity indicators with type 2 diabetes risk: two large prospective studies in US men and women. *Eur J Epidemiol*. (2018) 33:1113–23. 10.1007/s10654-018-0433-5 30117031

[16] Liu M Zhang Z Zhou C Ye Z He P Zhang Y et al. Predicted fat mass and lean mass in relation to all-cause and cause-specific mortality. *J Cachexia Sarcopenia Muscle.* (2022) 13:1064–75. 10.1002/jcsm.12921 35068076 PMC8978015

[17] Fan JG. Chinese guidelines for the diagnosis and management of nonalcoholic fatty liver disease (2010 updated edition) [In Chinese]. *Chin J Clin Med Front.* (2012) 4:4–10. 25372639

[18] Liu Y Shuai P Chen W Liu Y Li D. Association between *Helicobacter pylori* infection and metabolic syndrome and its components. *Front Endocrinol*. (2022) 14:1188487. 10.3389/fendo.2023.1188487 37404306 PMC10316390

[19] Chinese Hypertension League, National Center for Cardiovascular Diseases. Chinese guidelines for the prevention and treatment of hypertension (2018 revised edition) [In Chinese]. *Chin J Cardiovasc Med.* (2019) 24:24–56.

[20] Ye C Kong L Wang Y Zheng J Xu M Xu Y et al. Causal associations of sarcopenia-related traits with cardiometabolic disease and Alzheimer’s disease and the mediating role of insulin resistance: a Mendelian randomization study. *Aging Cell*. (2023) 22:e13923. 10.1111/acel.13923 37403750 PMC10497819

[21] Peng TC Wu LW Chen WL Liaw FY Chang YW Kao TW. Nonalcoholic fatty liver disease and sarcopenia in a Western population (NHANES III): the importance of sarcopenia definition. *Clin Nutr* (2019) 38:422–8. 10.1016/j.clnu.2017.11.021 29287676

[22] Ahmed B Sultana R Greene MW. Adipose tissue and insulin resistance in obese. *Biomed Pharmacother.* (2021) 137:111315. 10.1016/j.biopha.2021.111315 33561645

[23] Carranza-Trejo AM Vetvicka V Vistejnova L Kralickova M Montufar EB. Hepatocyte and immune cell crosstalk in non-alcoholic fatty liver disease. *Expert Rev Gastroenterol Hepatol*. (2021) 15:783–96. 10.1080/17474124.2021.1887730 33557653

[24] Bhanji RA Narayanan P Allen AM Malhi H Watt KD. Sarcopenia in hiding: the risk and consequence of underestimating muscle dysfunction in nonalcoholic steatohepatitis. *Hepatology*. (2017) 66:2055–65. 10.1002/hep.29420 28777879

