# Supplementary material for: Association between predicted body composition and metabolic-associated fatty liver disease: a case-control study in a Chinese population
Source: Front Med (Lausanne). 2026 Jun 10;13:1750432. doi: 10.3389/fmed.2026.1750432 (PMC13290577; doi:10.3389/fmed.2026.1750432)
Supplement: Supplementary file 1 [file Data_Sheet_1.docx]

Table S1 Anthropometric prediction equation

| Equation profiles |  |
| --- | --- |
| Equation 1  Male LBM (kg) = -14.729 - 0.071*age (year) + 0.210*height (cm) + 0.468*weight (kg) - 0.441 *Mexican + 0.320 *Hispanic + 1.821*Black – 0.784*other ethnicity | R^2^ = 0.88，SEE = 2.96 kg |
| Equation 2  Female LBM (kg) = -14.292 - 0.046*age (year) + 0.201*height (cm) + 0.347*weight (kg) - 0.448 *Mexican - 0.047 *Hispanic + 1.128*Black – 0.384*other ethnicity | R^2^ = 0.85，SEE = 2.39 kg |
| Equation 3  Male FM (kg) = 17.391 + 0.068*age (year) - 0.234*height (cm) + 0.530*weight (kg) + 0.477 *Mexican -0.282 *Hispanic - 1.949*Black + 0.815*other ethnicity | R^2^ = 0.86，SEE = 3.05 kg |
| Equation 4  Female FM (kg) = 15.513 + 0.048*age (year) - 0.215*height (cm) + 0.646*weight (kg) + 0.479 *Mexican + 0.061*Hispanic – 1.230*Black + 0.370*other ethnicity | R^2^ = 0.93，SEE = 2.45kg |
| Equation 5  Male BF% = 44.47 + 0.10*age (year) - 0.26*height (cm) + 0.29*weight (kg) + 0.81 *Mexican - 0.09*Hispanic - 0.246*Black + 0.82*other ethnicity | R^2^ = 0.61，SEE = 3.61% |
| Equation 6  Female BF% = 58.60 + 0.08*age (year) - 0.30*height (cm) + 0.35*weight (kg) + 1.09 *Mexican + 0.46*Hispanic - 1.66*Black - 0.53*other ethnicity | R^2^ = 0.64，SEE = 3.87% |

Table S2 BMI grouping analysis results of FM, BF%, LBM, FM/LBM and risk of MASLD

| Variables | BMI＜24 | |  | 24≤BMI＜28 | |  | BMI≥28 | |
| --- | --- | --- | --- | --- | --- | --- | --- | --- |
|  | *OR (95%CI)* | *P* |  | *OR (95%CI)* | *P* |  | *OR (95%CI)* | *P* |
| FM^ac^ |  |  |  |  |  |  |  |  |
| Q1 | 1.00 (ref.) |  |  | 1.00 (ref.) |  |  | 1.00 (ref.) |  |
| Q2 | 1.83(1.20-2.80) | 0.005 |  | 1.25(0.99-1.97) | 0.056 |  | 1.07(0.78-1.48) | 0.673 |
| Q3 | 2.55(1.69-3.84) | <0.001 |  | 1.57(1.21-2.04) | 0.001 |  | 1.10(0.80-1.51) | 0.547 |
| Q4 | 4.49(2.04-6.64) | <0.001 |  | 1.89(1.33-2.68) | <0.001 |  | 1.24(0.89-1.73) | 0.208 |
| Trend test |  | <0.001 |  |  | 0.001 |  |  | 0.199 |
| BF%^c^ |  |  |  |  |  |  |  |  |
| Q1 | 1.00 (ref.) |  |  | 1.00 (ref.) |  |  | 1.00 (ref.) |  |
| Q2 | 2.10(1.47-3.02) | <0.001 |  | 1.22(0.98-1.51) | 0.071 |  | 1.12(0.81-1.54) | 0.490 |
| Q3 | 2.66(1.83-3.86) | <0.001 |  | 1.36(1.07-2.74) | 0.013 |  | 1.17(0.83-1.65) | 0.378 |
| Q4 | 6.08(3.78-9.80) | <0.001 |  | 1.73(1.22-2.46) | 0.002 |  | 1.29(0.90-1.82) | 0.171 |
| Trend test |  | <0.001 |  |  | 0.009 |  |  | 0.213 |
| LBM^ab^ |  |  |  |  |  |  |  |  |
| Q1 | 1.00 (ref.) |  |  | 1.00 (ref.) |  |  | 1.00 (ref.) |  |
| Q2 | 1.25(0.86-1.83) | 0.248 |  | 1.06(0.75-1.50) | 0.745 |  | 1.04(0.70-1.49) | 0.841 |
| Q3 | 2.03(1.26-3.27) | 0.004 |  | 1.16(0.78-1.73) | 0.476 |  | 0.96(0.63-1.48) | 0.863 |
| Q4 | 2.55(1.41-4.58) | 0.002 |  | 1.11(0.67-1.81) | 0.693 |  | 0.98(0.55-1.76) | 0.958 |
| Trend test |  | <0.001 |  |  | 0.622 |  |  | 0.886 |
| FM/LBM |  |  |  |  |  |  |  |  |
| Q1 | 1.00 (ref.) |  |  | 1.00 (ref.) |  |  | 1.00 (ref.) |  |
| Q2 | 2.35(1.62-3.42) | <0.001 |  | 1.22(0.99-1.51) | 0.062 |  | 1.11(0.81-1.54) | 0.513 |
| Q3 | 2.68(1.83-3.91) | <0.001 |  | 1.44(1.14-1.81) | 0.002 |  | 1.15(0.82-1.62) | 0.424 |
| Q4 | 3.45(2.32-5.12) | <0.001 |  | 1.78(1.28-2.46) | 0.001 |  | 1.28(0.90-1.83) | 0.176 |
| Trend test |  | <0.001 |  |  | 0.005 |  |  | 0.221 |

Note: Multi-factor adjustment model. Adjusted for ethnicity, educational level, smoking history, drinking history, tea consumption history, weekly exercise, sedentary time, hypertension, hyperglycemia, and uric acid. FM: Fat mass; BF% : Fat percentage; LBM: Lean body mass; FM/LBM: The ratio of fat mass to lean body mass.

^a^ corrected the height. ^b^ further corrected FM. ^c^ further corrected LBM.

Table S3 Age grouping analysis results of FM, BF%, LBM, FM/LBM and risk of MASLD

| Variables | ＜40 years | |  | 40-60 years | |  | ≥60 years | |
| --- | --- | --- | --- | --- | --- | --- | --- | --- |
|  | *OR (95%CI)* | *P* |  | *OR (95%CI)* | *P* |  | *OR (95%CI)* | *P* |
| FM^ac^ |  |  |  |  |  |  |  |  |
| Q1 | 1.00 (ref.) |  |  | 1.00 (ref.) |  |  | 1.00 (ref.) |  |
| Q2 | 2.62(1.88-3.67) | <0.001 |  | 1.55(1.19-2.04) | 0.001 |  | 1.53(0.69-3.41) | 0.297 |
| Q3 | 3.76(2.70-5.24) | <0.001 |  | 1.86(1.42-2.42) | <0.001 |  | 2.23(1.05-4.74) | 0.036 |
| Q4 | 4.19(2.95-5.93) | <0.001 |  | 2.21(1.68-2.90) | <0.001 |  | 2.28(1.01-5.11) | 0.046 |
| Trend test |  | <0.001 |  |  | <0.001 |  |  | 0.042 |
| BF%^c^ |  |  |  |  |  |  |  |  |
| Q1 | 1.00 (ref.) |  |  | 1.00 (ref.) |  |  | 1.00 (ref.) |  |
| Q2 | 2.39(1.78-3.22) | <0.001 |  | 1.70(1.32-2.17) | <0.001 |  | 1.77(0.84-3.79) | 0.132 |
| Q3 | 3.15(2.36-4.21) | <0.001 |  | 1.79(1.39-2.30) | <0.001 |  | 2.13(1.03-4.38) | 0.040 |
| Q4 | 3.12(1.33-4.17) | <0.001 |  | 2.20(1.70-2.91) | <0.001 |  | 2.00(0.97-4.13) | 0.061 |
| Trend test |  | <0.001 |  |  | <0.001 |  |  | 0.293 |
| LBM^ab^ |  |  |  |  |  |  |  |  |
| Q1 | 1.00 (ref.) |  |  | 1.00 (ref.) |  |  | 1.00 (ref.) |  |
| Q2 | 1.29(0.97-1.73) | 0.084 |  | 1.14(0.87-1.51) | 0.346 |  | 1.15(0.56-2.33) | 0.706 |
| Q3 | 1.93(1.43-2.60) | <0.001 |  | 1.43(1.06-1.93) | 0.020 |  | 1.39(0.62-3.14) | 0.428 |
| Q4 | 1.89(1.32-2.71) | 0.001 |  | 1.47(1.03-2.08) | 0.032 |  | 1.68(0.65-4.36) | 0.287 |
| Trend test |  | <0.001 |  |  | 0.018 |  |  | 0.301 |
| FM/LBM |  |  |  |  |  |  |  |  |
| Q1 | 1.00 (ref.) |  |  | 1.00 (ref.) |  |  | 1.00 (ref.) |  |
| Q2 | 2.79(2.05-3.80) | <0.001 |  | 1.68(1.31-2.16) | <0.001 |  | 1.87(0.90-3.88) | 0.092 |
| Q3 | 3.61(2.67-4.87) | <0.001 |  | 1.89(1.48-2.42) | <0.001 |  | 2.16(1.08-4.34) | 0.030 |
| Q4 | 3.69(2.73-4.99) | <0.001 |  | 1.97(1.52-2.56) | <0.001 |  | 1.98(0.96-4.08) | 0.065 |
| Trend test |  | <0.001 |  |  | <0.001 |  |  | 0.207 |

Note: Multi-factor adjustment model. Adjusted for ethnicity, educational level, smoking history, drinking history, tea consumption history, weekly exercise, sedentary time, hypertension, hyperglycemia, and uric acid. FM: Fat mass; BF% : Fat percentage; LBM: Lean body mass; FM/LBM: The ratio of fat mass to lean body mass.

^a^ corrected the height. ^b^ further corrected FM. ^c^ further corrected LBM.

Table S4 Hypertension grouping analysis results of FM, BF%, LBM, FM/LBM and risk of MASLD

| Variables | Hypertension group | |  | Non-hypertension group | |
| --- | --- | --- | --- | --- | --- |
|  | *OR (95%CI)* | *P* |  | *OR (95%CI)* | *P* |
| FM^ac^ |  |  |  |  |  |
| Q1 | 1.00 (ref.) |  |  | 1.00 (ref.) |  |
| Q2 | 1.54(1.05-2.26) | 0.027 |  | 2.20(1.74-2.78) | <0.001 |
| Q3 | 1.73(1.17-2.53) | 0.005 |  | 2.83(2.25-3.56) | <0.001 |
| Q4 | 1.81(1.19-2.76) | 0.006 |  | 3.36(2.65-4.26) | <0.001 |
| Trend test |  | 0.012 |  |  | <0.001 |
| BF%^c^ |  |  |  |  |  |
| Q1 | 1.00 (ref.) |  |  | 1.00 (ref.) |  |
| Q2 | 1.45(1.00-2.11) | 0.050 |  | 1.90(1.55-2.33) | <0.001 |
| Q3 | 1.49(1.01-2.21) | 0.045 |  | 2.29(1.88-2.78) | <0.001 |
| Q4 | 1.50(1.00-2.23) | 0.048 |  | 2.70(2.21-3.31) | <0.001 |
| Trend test |  | 0.237 |  |  | <0.001 |
| LBM^ab^ |  |  |  |  |  |
| Q1 | 1.00 (ref.) |  |  | 1.00 (ref.) |  |
| Q2 | 1.44(0.96-2.15) | 0.076 |  | 1.14(0.92-1.42) | 0.223 |
| Q3 | 1.44(0.93-2.23) | 0.100 |  | 1.67(1.33-2.09) | <0.001 |
| Q4 | 1.39(0.81-2.39) | 0.231 |  | 1.74(1.33-2.29) | <0.001 |
| Trend test |  | 0.177 |  |  | <0.001 |
| FM/LBM |  |  |  |  |  |
| Q1 | 1.00 (ref.) |  |  | 1.00 (ref.) |  |
| Q2 | 1.73(1.20-2.50) | 0.003 |  | 1.75(1.15-2.65) | <0.001 |
| Q3 | 1.79(1.24-2.57) | 0.002 |  | 2.74(2.23-3.39) | <0.001 |
| Q4 | 1.84(1.26-2.69) | 0.002 |  | 3.07(2.47-3.82) | <0.001 |
| Trend test |  | 0.023 |  |  | <0.001 |

Note: Multi-factor adjustment model. Adjusted for ethnicity, educational level, smoking history, drinking history, tea consumption history, weekly exercise, sedentary time, hypertension, hyperglycemia, and uric acid. FM: Fat mass; BF% : Fat percentage; LBM: Lean body mass; FM/LBM: The ratio of fat mass to lean body mass.

^a^ corrected the height. ^b^ further corrected FM. ^c^ further corrected LBM.

Table S5 Gender grouping analysis results of FM, BF%, LBM, FM/LBM and risk of MASLD

| Variables | Male | |  | female | |
| --- | --- | --- | --- | --- | --- |
|  | *OR (95%CI)* | *P* |  | *OR (95%CI)* | *P* |
| FM^ac^ |  |  |  |  |  |
| Q1 | 1.00 (ref.) |  |  | 1.00 (ref.) |  |
| Q2 | 2.10(1.65-2.68) | <0.001 |  | 2.05(1.29-3.24) | 0.002 |
| Q3 | 2.64(2.04-3.41) | <0.001 |  | 3.16(1.96-5.09) | <0.001 |
| Q4 | 2.77(2.01-3.80) | <0.001 |  | 3.03(1.66-5.56) | <0.001 |
| Trend test |  | <0.001 |  |  | <0.001 |
| BF%^c^ |  |  |  |  |  |
| Q1 | 1.00 (ref.) |  |  | 1.00 (ref.) |  |
| Q2 | 1.92(1.54-2.38) | <0.001 |  | 1.76(1.16-2.68) | 0.008 |
| Q3 | 2.59(2.11-3.20) | <0.001 |  | 2.84(1.88-4.28) | <0.001 |
| Q4 | 3.06(2.48-3.79) | <0.001 |  | 3.18(2.06-4.92) | <0.001 |
| Trend test |  | <0.001 |  |  | <0.001 |
| LBM^ab^ |  |  |  |  |  |
| Q1 | 1.00 (ref.) |  |  | 1.00 (ref.) |  |
| Q2 | 1.54(1.23-1.91) | <0.001 |  | 1.58(1.05-2.36) | 0.027 |
| Q3 | 1.86(1.46-2.36) | <0.001 |  | 1.74(1.10-2.76) | 0.019 |
| Q4 | 1.83(1.34-2.51) | <0.001 |  | 2.05(1.09-3.87) | 0.026 |
| Trend test |  | <0.001 |  |  | 0.038 |
| FM/LBM |  |  |  |  |  |
| Q1 | 1.00 (ref.) |  |  | 1.00 (ref.) |  |
| Q2 | 2.02(1.62-2.51) | <0.001 |  | 1.78(1.17-2.72) | 0.007 |
| Q3 | 2.70(2.19-3.33) | <0.001 |  | 2.99(1.99-4.47) | <0.001 |
| Q4 | 3.11(2.51-3.84) | <0.001 |  | 3.48(2.31-5.24) | <0.001 |
| Trend test |  | <0.001 |  |  | <0.001 |

Note: Multi-factor adjustment model. Adjusted for ethnicity, educational level, smoking history, drinking history, tea consumption history, weekly exercise, sedentary time, hypertension, hyperglycemia, and uric acid. FM: Fat mass; BF%: Fat percentage; LBM: Lean body mass; FM/LBM: The ratio of fat mass to lean body mass.

^a^ corrected the height. ^b^ further corrected FM. ^c^ further corrected LBM.

Table S6 hyperglycemia grouping analysis results of FM, BF%, LBM, FM/LBM and risk of MASLD

| Variables | hyperglycemia | |  | Non-hyperglycemia | |
| --- | --- | --- | --- | --- | --- |
|  | *OR (95%CI)* | *P* |  | *OR (95%CI)* | *P* |
| FM^ac^ |  |  |  |  |  |
| Q1 | 1.00 (ref.) |  |  | 1.00 (ref.) |  |
| Q2 | 1.68(0.87-3.24) | 0.126 |  | 2.10(1.70-2.58) | <0.001 |
| Q3 | 1.71(0.89-3.28) | 0.106 |  | 2.72(2.22-3.32) | <0.001 |
| Q4 | 1.88(0.89-3.96) | 0.096 |  | 3.35(2.73-4.10) | <0.001 |
| Trend test |  | 0.135 |  |  | <0.001 |
| BF%^c^ |  |  |  |  |  |
| Q1 | 1.00 (ref.) |  |  | 1.00 (ref.) |  |
| Q2 | 1.66(0.93-2.98) | 0.088 |  | 1.79(1.49-2.15) | <0.001 |
| Q3 | 1.44(0.79-2.62) | 0.236 |  | 2.15(1.80-2.58) | <0.001 |
| Q4 | 1.88(1.05-3.35) | 0.033 |  | 2.43(2.02-2.92) | <0.001 |
| Trend test |  | 0.076 |  |  | <0.001 |
| LBM^ab^ |  |  |  |  |  |
| Q1 | 1.00 (ref.) |  |  | 1.00 (ref.) |  |
| Q2 | 0.95(0.47-1.94) | 0.894 |  | 1.02(0.84-1.23) | 0.866 |
| Q3 | 1.17(0.56-2.42) | 0.694 |  | 1.27(1.07-1.52) | 0.008 |
| Q4 | 0.99(0.40-2.49) | 0.986 |  | 1.14(0.95-1.36) | 0.163 |
| Trend test |  | 0.903 |  |  | 0.079 |
| FM/LBM |  |  |  |  |  |
| Q1 | 1.00 (ref.) |  |  | 1.00 (ref.) |  |
| Q2 | 1.75(0.94-3.26) | 0.080 |  | 1.91(1.59-2.31) | <0.001 |
| Q3 | 2.70(0.79-3.33) | 0.124 |  | 2.37(1.97-2.84) | <0.001 |
| Q4 | 2.14(1.11-3.15) | 0.024 |  | 2.45(2.03-2.95) | <0.001 |
| Trend test |  | 0.046 |  |  | <0.001 |

Note: Multi-factor adjustment model. Adjusted for ethnicity, educational level, smoking history, drinking history, tea consumption history, weekly exercise, sedentary time, hypertension, hyperglycemia, and uric acid. FM: Fat mass; BF% : Fat percentage; LBM: Lean body mass; FM/LBM: The ratio of fat mass to lean body mass.

^a^ corrected the height. ^b^ further corrected FM. ^c^ further corrected LBM.

Table S7. Frequency of medication use in the matched study population

| Medication use | MASLD group (n=1683) | Control group (n=1683) |
| --- | --- | --- |
| Glucose-lowering agents | 1 (0.06%) | 0 (0.00%) |
| Antihypertensive agents | 0 (0.00%) | 2 (0.12%) |
| Lipid-lowering agents | 0 (0.00%) | 1 (0.06%) |

Note: Due to extremely low frequencies, statistical comparison was not performed.

Table S8. Incremental predictive value of body composition indices for MASLD compared with BMI

| Model | AUC | 95% CI | ΔAUC | *P* value* |
| --- | --- | --- | --- | --- |
| Age + sex + BMI | 0.796 | 0.782-0.810 | reference | - |
| + FM | 0.797 | 0.783-0.811 | 0.001 | 0.299 |
| + BF% | 0.796 | 0.782-0.810 | 0.000 | 0.955 |
| + LBM | 0.797 | 0.783-0.811 | 0.001 | 0.218 |
| + FM/LBM | 0.796 | 0.782-0.810 | 0.000 | 0.984 |

*DeLong test for comparison with the base model.

Table S9. Subgroup-specific AUCs of body composition indices for MASLD prediction

| Subgroup | Index | AUC | 95% CI |
| --- | --- | --- | --- |
| Non-obese (BMI<24)  (n=1501) | FM | 0.702 | 0.678–0.725 |
|  | BF% | 0.629 | 0.604–0.654 |
|  | LBM | 0.539 | 0.514–0.565 |
|  | FM/LBM | 0.643 | 0.618–0.667 |
| Obese (BMI≥28)  (n=439) | FM | 0.643 | 0.597–0.688 |
|  | BF% | 0.623 | 0.576–0.668 |
|  | LBM | 0.524 | 0.476–0.572 |
|  | FM/LBM | 0.615 | 0.568–0.661 |
| Age <60  (n=3123) | FM | 0.782 | 0.767–0.796 |
|  | BF% | 0.680 | 0.664–0.697 |
|  | LBM | 0.661 | 0.644–0.678 |
|  | FM/LBM | 0.694 | 0.677–0.710 |
| Age ≥60  (n=243) | FM | 0.789 | 0.732–0.839 |
|  | BF% | 0.683 | 0.620–0.741 |
|  | LBM | 0.684 | 0.622–0.742 |
|  | FM/LBM | 0.685 | 0.623–0.743 |

Note: AUCs were calculated using single predictors without covariate adjustment, following the same method as in Table 4.

Table S10. Sensitivity analysis: Associations of body composition indices with MASLD after additional adjustment for BMI

| Variables | Original Model (Model 3) | Sensitivity Model (Model 3 + BMI) |
| --- | --- | --- |
| FM (Q4 vs Q1) | OR=2.77 (2.26–3.40) | OR=1.05 (0.51–2.18) |
| BF% (Q4 vs Q1) | OR=2.16 (1.78–2.62) | OR=1.68 (0.85–3.31) |
| LBM (Q4 vs Q1) | OR=1.62 (1.27–2.04) | OR=1.32 (0.56–3.11) |
| FM/LBM (Q4 vs Q1) | OR=2.37 (1.99–2.83) | OR=2.00 (1.43–2.78) |

Note: After additional adjustment for BMI, FM, BF%, and LBM were no longer statistically significant (all *P* > 0.05), whereas FM/LBM remained significant (*P* < 0.05). This is expected because FM/LBM reflects the relative balance between fat and lean mass, providing information independent of overall adiposity captured by BMI.
